# Dedifferentiated parosteal osteosarcoma of the maxilla: a case report and review of the literature

**DOI:** 10.1186/s13256-018-1747-3

**Published:** 2018-08-17

**Authors:** Hidetaka Miyashita, Kazunari Yoshida, Tomoya Soma, Kaori Kameyama, Aya Sasaki, Masanori Hisaoka, Masaki Yazawa, Hideo Morioka, Moe Takahashi, Taneaki Nakagawa, Hiromasa Kawana

**Affiliations:** 1 Department of Dentistry and Oral Surgery, Division of Oral and Maxillofacial Surgery, Keio University, School of Medicine, 35 Shinanomachi, Shinjuku-ku, Tokyo, 160-8582 Japan; 2 Department of Neurosurgery, Keio University School of Medicine, 35 Shinanomachi, Shinjuku-ku, Tokyo, 160-8582 Japan; 3 Department of Diagnostic Pathology, Keio University School of Medicine, 35 Shinanomachi, Shinjuku-ku, Tokyo, 160-8582 Japan; 4 Department of Pathology and Oncology, University of Occupational and Environmental Health, 1-1 Iseigaoka, Yahatanishi-ku, Kitakyushu, 807-8555 Japan; 5 Department of Plastic and Reconstructive Surgery, Keio University School of Medicine, 35 Shinanomachi, Shinjuku-ku, Tokyo, 160-8582 Japan; 6 Department of Orthopaedic Surgery, Keio University, School of Medicine, 35 Shinanomachi, Shinjuku-ku, Tokyo, 160-8582 Japan

**Keywords:** Parosteal osteosarcoma, Dedifferentiated, Maxilla, Head and neck, Chemotherapy, Case report

## Abstract

**Background:**

Parosteal osteosarcomas are usually low-grade tumors, however, sometimes they transform to high-grade tumors, which is named dedifferentiation. This phenomenon has been reported in long bones. Recently, we encountered a patient with dedifferentiated parosteal osteosarcoma occurring in the maxilla. Here, we report a first case of dedifferentiated parosteal osteosarcoma of the head and neck region.

**Case presentation:**

A 45-year-old Japanese woman with a refractory bone lesion in the maxilla presented to our hospital. A biopsy showed atypical spindle cell proliferation involving dedifferentiated high-grade component, which was diagnosed as dedifferentiated parosteal osteosarcoma. Three cycles of neoadjuvant chemotherapy using ifosfamide and pirarubicin were performed followed by sub-total maxillectomy. Histopathological results showed that neoadjuvant chemotherapy was effective for high-grade component. The decision to perform adjuvant chemotherapy (cisplatin and pirarubicin) was made because distant metastasis has been reported, even in cases with dedifferentiated parosteal osteosarcoma in which complete necrosis of high-grade component was achieved due to neoadjuvant chemotherapy. There was no recurrence 15 months after surgery.

**Conclusions:**

Dedifferentiated parosteal osteosarcoma can occur in the head and neck region. Chemotherapy including anthracycline anticancer agent could be effective for high-grade component of dedifferentiated parosteal osteosarcoma.

## Background

Dedifferentiated parosteal osteosarcoma (DPOS) is defined as a high-grade surface osteosarcoma which rarely occurs as either a primary or secondary event of conventional low-grade parosteal osteosarcoma (c-POS) [1]. Several incidences of dedifferentiation in c-POS were reported by earlier studies (18–24%) [1–3]. c-POS is known to have a good prognosis after local excision. However, DPOS has the ability to metastasize systemically, which results in an uncontrollable condition [4]. Although many treatment strategies have been attempted, definite evidence-based treatment has not yet been established due to a low incidence rate of DPOS. DPOS mainly arises from long bones such as femur, humerus, and tibia [3]. However, to the best of our knowledge, cases of DPOS arising from the head and neck region have not been reported so far. Here, we present a case of DPOS of the maxilla treated with combination therapy, including surgery and chemotherapy. Furthermore, we review the available literature and we discuss treatment strategies with a focus on chemotherapy.

## Case presentation

**Fig. 1:**
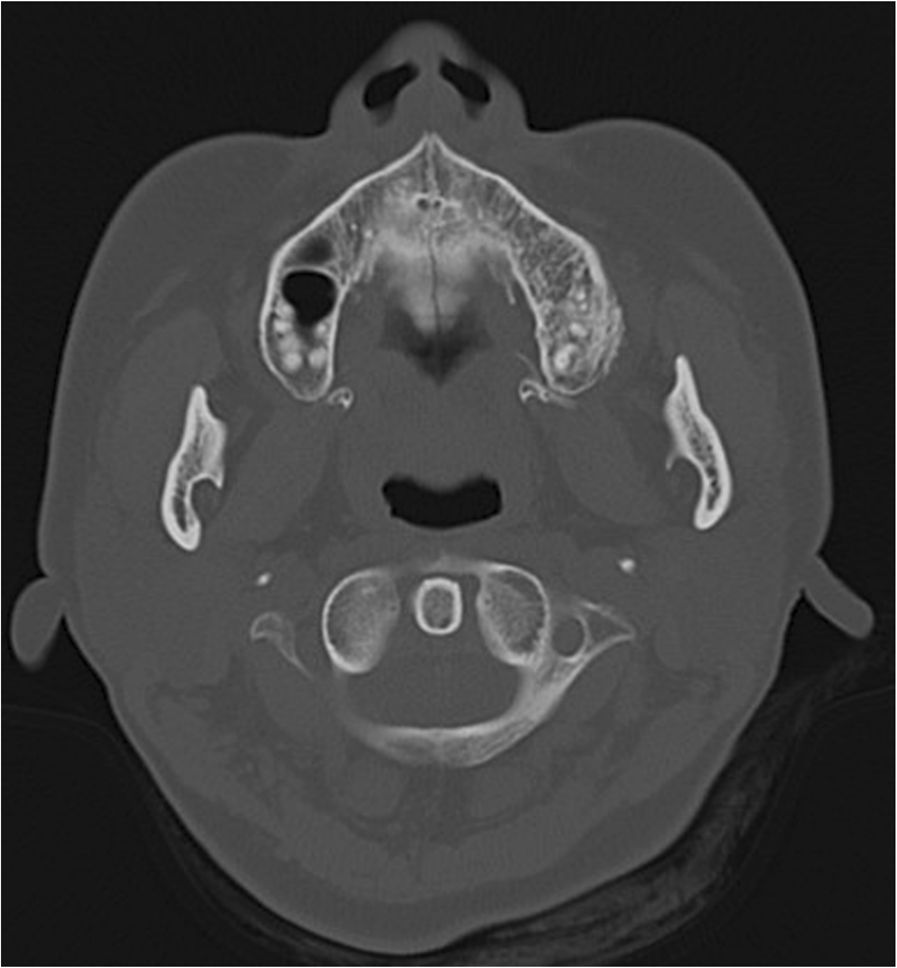
Computed tomography image at the first visit showing diffuse radiopaque lesion arising from upper left molar region

**Fig. 2:**
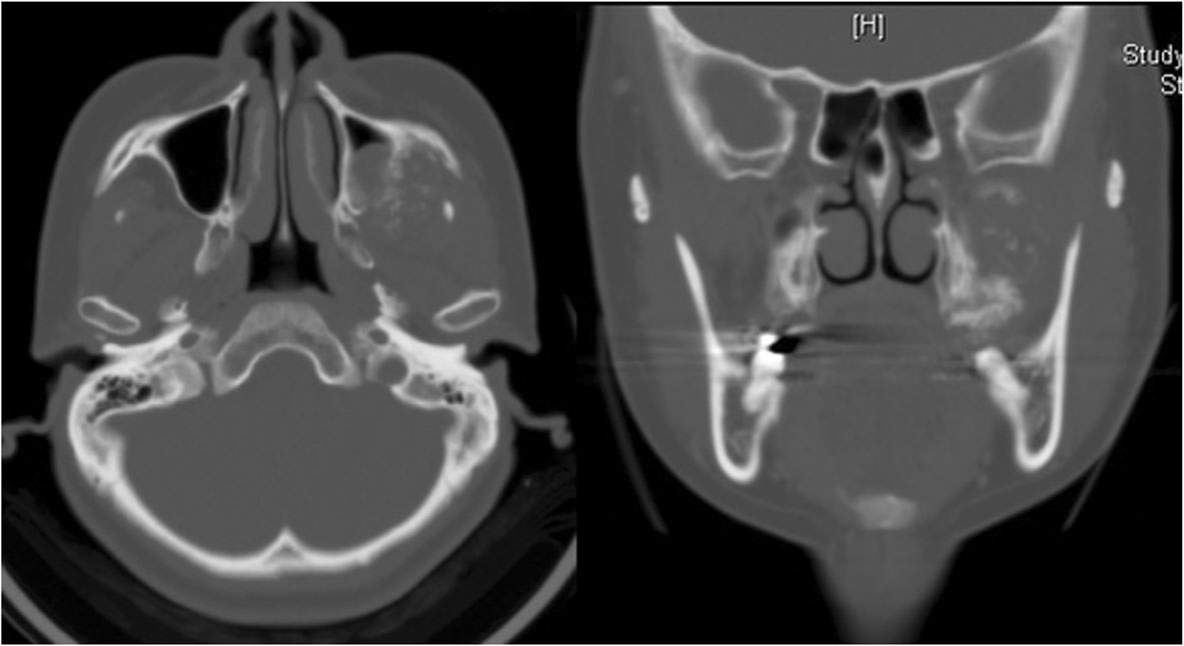
Computed tomography image showing heterogeneous bone mass. Feasibility of medullary bone invasion toward the inferior orbital fissure via pterygopalatine fossa

**Fig. 3:**
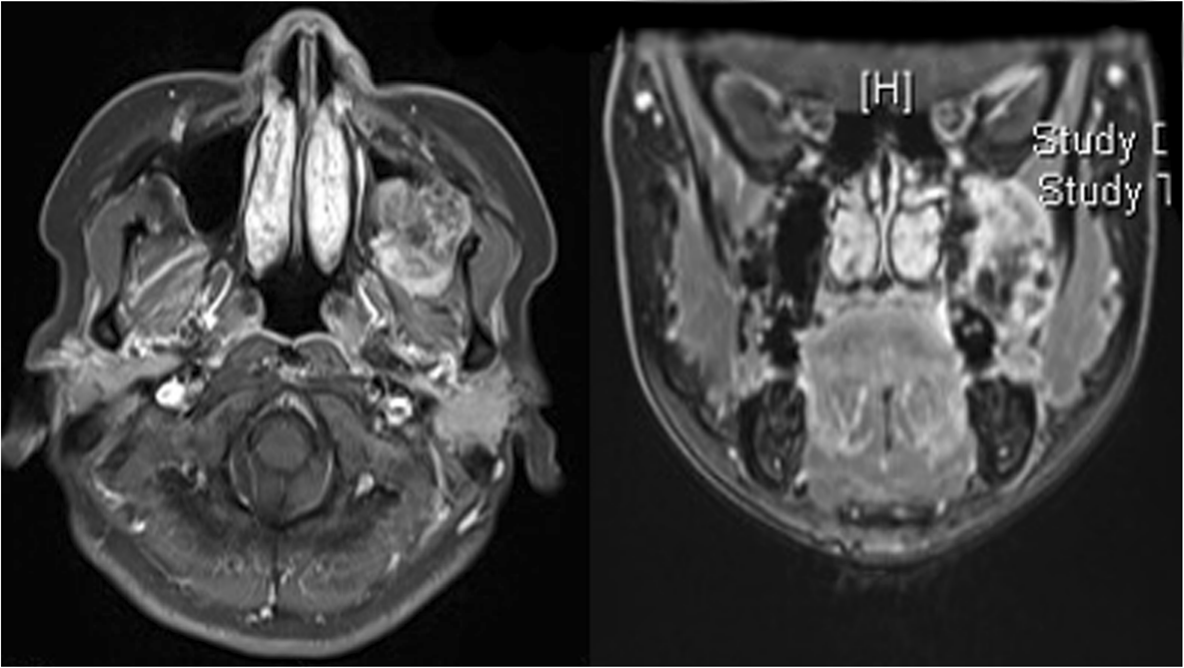
Magnetic resonance imaging showing diffuse mass with the contrast effect existing mainly in the pterygopalatine fossa. The contrast-enhanced areas correspond with the radiolucent area on computed tomography

**Fig. 4:**
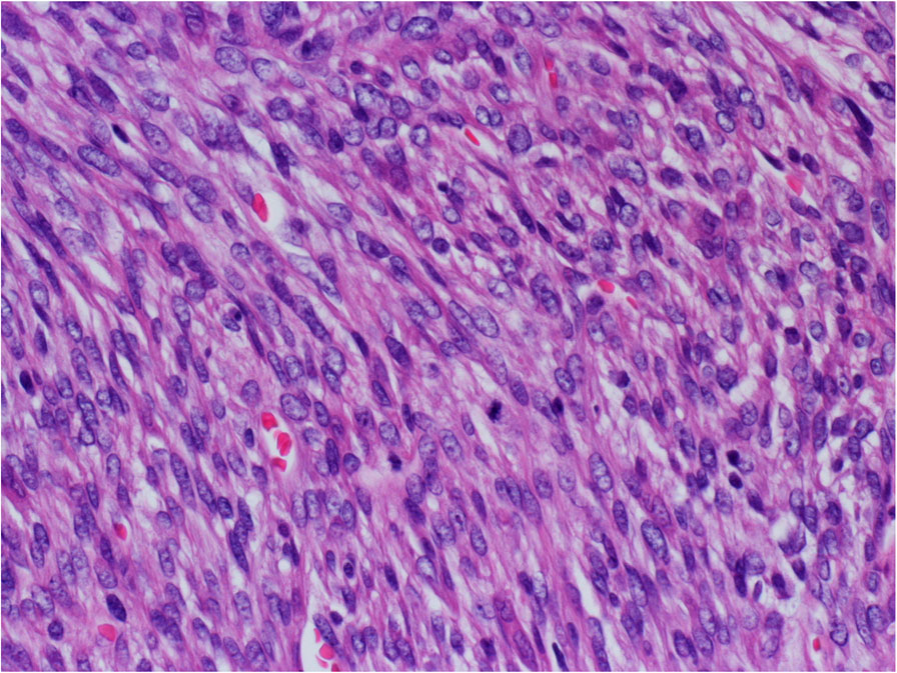
Hematoxylin-eosin staining demonstrating the atypical spindle cells with nuclear enlargement and nucleus with irregular shape

**Fig. 5:**
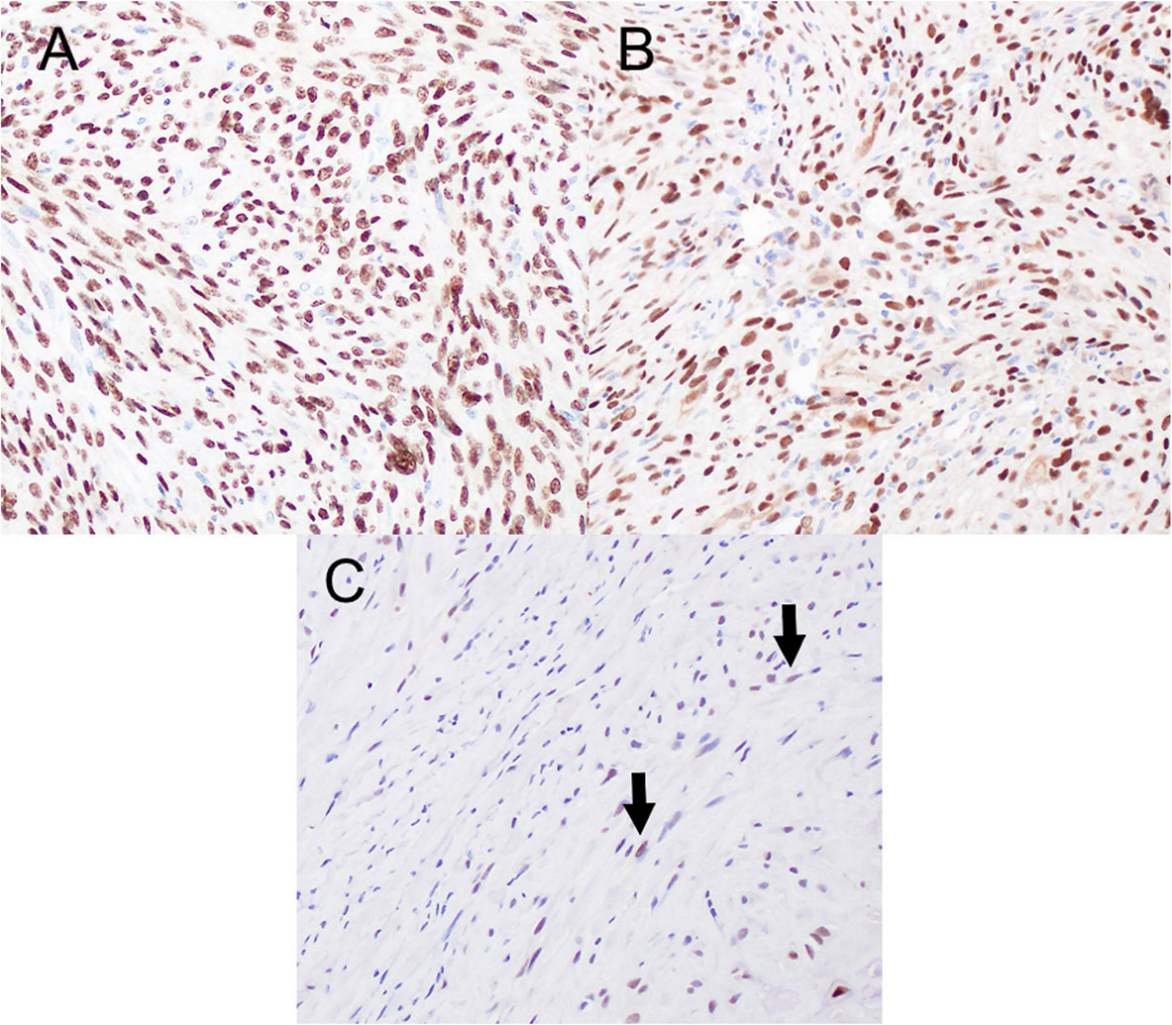
Immunohistochemical staining findings demonstrating the origin of atypical spindle cells. **a**, **b** Positive findings of RUNX-2 and SATB2 staining osteoblastic cell. **c** SOX-9 positive indicated by *arrows* suggests the origin of these spindle cells from cartilage cells

A 45-year-old Japanese woman with a swelling and bone exposure of the left buccal region was referred to our hospital. She had previously undergone excisional biopsy two times, which led to the same diagnosis of osteoma. She had no special medical and family history. On clinical examination, maxillary bone exposure without pain was observed around her upper left second molar. Other physical status was normal. Computed tomography (CT) showed a diffuse radiopaque lesion around the alveolar cortical bone surface of her maxilla spanning from the first molar to the second molar (Fig. 1). A biopsy demonstrated features of necrotic bone without atypia. The exposed region in her maxilla recovered with healthy oral mucosa naturally after the biopsy without any additional treatment. However, she noticed bone exposure again in the same region after a year and swelling that tended to enlarge over time. As she did not want to undergo radical surgery requiring tooth extraction, we performed debulking surgery including biopsy twice in 2 years. However, these biopsies did not demonstrate features of malignancy. The lesion enlarged gradually during the observation period and CT showed a diffuse bone mass accompanied by radiolucent areas, which arose from the surface of maxillary alveolar bone and invaded into the pterygopalatine fossa (Fig. 2). T1-weighted fat-suppressed magnetic resonance imaging (MRI) after injection of intravenously administered contrast medium showed heterogeneous contrast-enhanced masses and hypointense areas corresponding to mineralized areas on CT (Fig. 3). Histopathological assessment showed stromal component consisting of dense atypical spindle cell proliferation and focal cartilage formation with mild atypia (Fig. 4). Immunohistochemical staining showed diffuse expression of β-catenin and α-smooth muscle actin (α-SMA) in atypical spindle cells. These cells were also stained by runt-related gene 2 (RUNX-2), special AT-rich sequence-binding protein 2 (SATB2), or sex-determining region Y-box 9 (SOX9), indicating that these cells were derived from cells that had the ability to differentiate into osteoblastic cells and cartilage cells (Fig. 5). Moreover, co-expression of murine double minute 2 (MDM2) and cyclin-dependent kinase 4 (CDK4) along with a dedifferentiated subtype of high-grade sarcoma was seen [5]. We excluded a diagnosis of chondrosarcoma and high-grade osteosarcoma because these histopathological features are usually negative in these conditions. Together with these findings, we arrived at a diagnosis of DPOS derived from c-POS. Indication of distant metastasis was not documented. Finally, we diagnosed this patient as stage IIB DPOS based on Enneking staging system [6].

The condition was treated by wide resection and chemotherapy. Since evidence-based chemotherapy for DPOS has not yet been established, we performed neoadjuvant chemotherapy (NAC) consisting of pirarubicin (THP-ADM) and ifosfamide (IFO) based on clinical trials carried out by the Japan Clinical Oncology Group (JCOG) 0304 [7]. THP-ADM (30 mg/m^2^, days 1 and 2) and IFO (2 g/m^2^, days 1–5) were administered and mesna (400 mg/m^2^) was added at 4 and 8 hours after infusion of IFO. This regimen was repeated for three cycles every 3 weeks. The administration dose was decreased by 40% for the second and third cycles, because grade 4 neutropenia and grade 3 febrile neutropenia were observed in the initial cycle. Although slight shrinking of the tumor was observed in radiological examination after three cycles of chemotherapy, the majority of the tumor did not demonstrate any other changes.

**Fig. 6:**
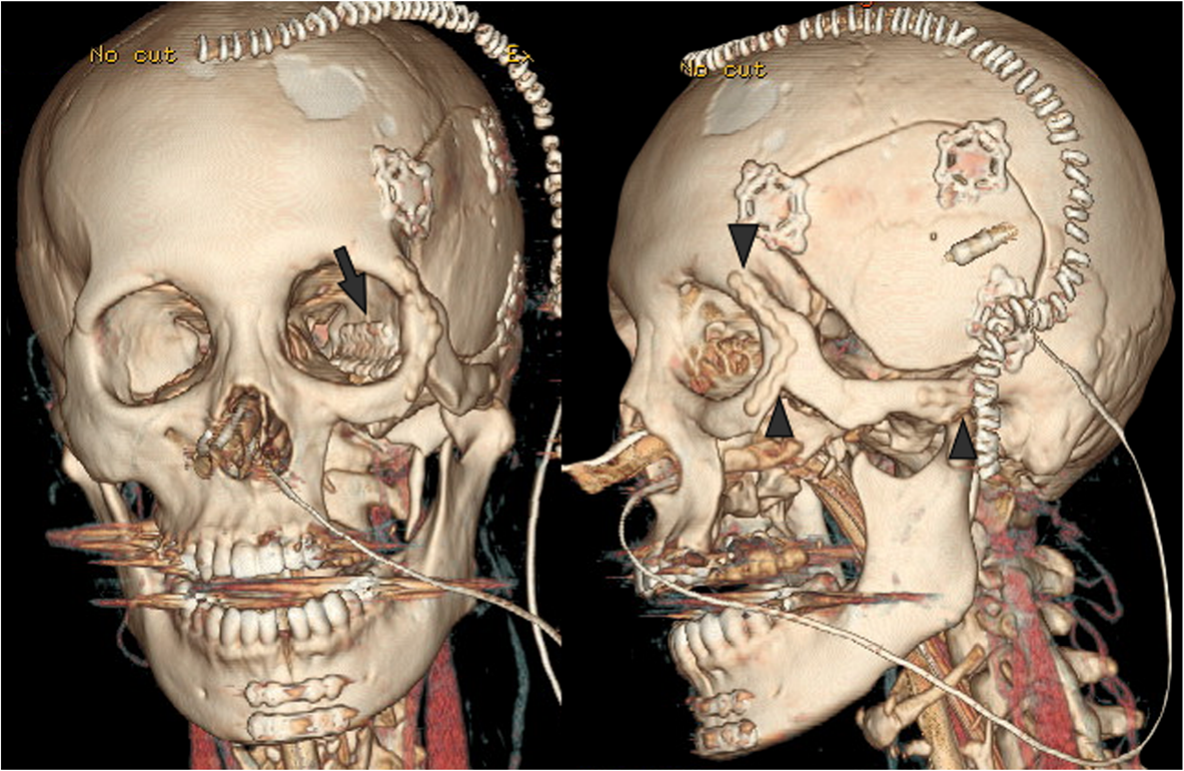
Postoperative computed tomography images. *Arrow* indicates titanium mesh applied on the lateral wall of the orbit. *Arrow heads* indicate the absorbent plate. The orbital rim and zygomatic arch were preserved

Subsequently, we performed subtotal maxillectomy using combined transcranial approach with orbitozygomatic osteotomy and transcervical approach with mandibulectomy. This approach was selected to avoid permanent surgical scar on our patient’s visible mid-face. Subtotal maxillectomy from her oral cavity was followed by a skull base surgery. Following resection of the lateral wall of the orbit, orbitozygomatic osteotomy was performed, which was contiguous to osteotomy from the oral cavity. The tumor invaded toward the foramen ovale and close to her optic nerve. We separated the tumor carefully from these anatomical structures on the upper levels of pterygopalatine fossa. Eventually, radical resection with an adequate margin was achieved followed by reconstruction surgery. After reconstruction of the lateral wall of the orbit with a titanium mesh, raw surface muscular flap reconstruction was also carried out immediately, as described previously [8]. Since morphological reconstruction mimicking natural morphology of the palate can be achieved by using this method, it was easier to apply the dental prosthesis for our patient. Finally, the temporal bone was restored and removed zygomatic bone was restored using an absorbent plate (Fig. 6).

The surgical specimen was examined histopathologically. Although a decrease in cell density and tumor degeneration due to NAC were observed in the high-grade component, the number of tumor cells in the low-grade component remained viable. Therefore, the chemotherapy effect was considered to be grade 1 based on the Huvos grading system (grade 1, 0–49% necrosis) [9]. Based on the recommendation of adjuvant chemotherapy in DPOS treatment [4], we decided to administer additional chemotherapy using cisplatin (CDDP) + THP-ADM. Adjuvant chemotherapy consisted of CDDP (120 mg/m^2^, day 1) and THP-ADM (30 mg/m^2^, days 1 and 2), and was repeated for two cycles every 3 weeks. The administration dose was decreased by 40% for all cycles due to experiences of adverse effects of NAC. The post-treatment progress was good, and no severe functional morbidity, such as eating dysfunction or dysphonia, was observed. As for our patient’s quality of life (QOL), her General Oral Health Assessment Indices (GOHAI) [10] were 59 and 53 points before operation and a year after surgery, respectively (generalized mean, 53.1; Table 1). She has shown no indication of recurrence after 15 months.

**Table 1:** General Oral Health Assessment Index scores

GOHAI items	Preoperation	A year after surgery
1. Limit the kinds of food	5	5
2. Trouble biting or chewing	5	3
3. Problems to swallow comfortably	5	5
4. Problems to speak clearly	5	3
5. Discomfort when eating any kind of food	5	4
6. Limit contact with people	5	5
7. Pleased with look of teeth	5	5
8. Used medication to relieve pain	5	5
9. Worried about teeth, gums or dentures	5	3
10. Self-conscious of teeth, gums or dentures	5	5
11. Uncomfortable eating in front of others	5	5
12. Sensitive to hot, cold or sweet foods	4	5
Total	59	53

Always – 1, Often – 2, Sometimes – 3, Seldom – 4, Never – 5. *GOHAI* General Oral Health Assessment Index

## Discussion

To the best of our knowledge, this is the first report of DPOS of the head and neck region. Previous literature has reported that 18% of cases of c-POS undergo dedifferentiation and cases of DPOS comprise a high-grade spindle cell sarcoma component coexisting with a lower grade typical parosteal osteosarcoma [2]. Dedifferentiation was often observed at the time of recurrence [1], although according to the experience of the Rizzoli Institute [3], the most dedifferentiated component was present in the initial lesion (synchronous). Therefore, in the case of c-POS diagnosed by biopsy before surgery, the potential for dedifferentiation occurring in primary c-POS should be considered in the treatment plan [3]. From the above viewpoints, accurate diagnostic methods for DPOS are needed to make appropriate treatment decisions. The usefulness of gadolinium (Gd)-enhanced T1-weighted MRI for detecting the dedifferentiation has been reported [11]. However, controversy exists over the value of MRI for the diagnosis of DPOS. Donmez *et al.* reported that MRI signal characteristics are not useful for tumor grading [12]. The main advantage of MRI is that contrast-enhanced images may delineate the appropriate biopsy site [12]. In the present case, we may have found the dedifferentiation by taking specimens from the contrast-enhanced region. It is challenging to make a diagnosis of DPOS before treatment. Comprehensive and careful assessments are required for an accurate diagnosis of DPOS.

Wide resection is generally accepted as the main treatment strategy for DPOS. However, the clinical importance of chemotherapy associated with wide resection surgical treatment is not clear. To the best of our knowledge, there are only three articles on DPOS in which the detailed histological assessment of preoperative chemotherapy is described (Table 2) [3, 4, 11]. Although the number of cases is small, NAC, including anthracycline anticancer agent, tends to be effective for DPOS. In the case presented here, we used IFO and THP-ADM for three cycles before surgery. Focusing on the high-grade component, almost all of these areas were necrotic due to the chemotherapy effect. The past literature [4], as described in Table 2, reported that the survival rate tends to be better in the group responding well to chemotherapy (with > 90% necrosis of the high-grade component). Moreover, disease-free survival was prolonged significantly (*P* = 0.03) in patients with a good response (median, 75 months) compared with those who responded poorly (median, 13 months) [4]. Together with these findings, chemotherapy including anthracycline anticancer agent could be effective for high-grade component of DPOS and may contribute to disease control. On the other hand, Bertoni *et al.* reported that there are no differences in survival rates between patients treated only with wide resection and wide resection with chemotherapy [3]. Furthermore, although c-POS has lower potential for distant metastasis, it cannot be ruled out that patients without dedifferentiation may develop distant metastasis [1]. In addition, Takeuchi *et al*. reported well-differentiated metastases in patients with DPOS, consisting of only the low-grade component and exhibiting a poor response to chemotherapy, thus concluding that dedifferentiation of tumors and the development of metastasis can occur independently [13]. In the present case, the chemotherapy effect was poor except for high-grade components. Given that DPOS coexists with c-POS, the value of chemotherapy for DPOS remains unclear. Further studies with evidence-based approaches are needed before chemotherapy treatment for DPOS can be standardized.

**Table 2:** Histopathological findings after preoperative chemotherapy

Reference	Number of cases	Sites	Surgery	Preoperative chemotherapy	Postoperative chemotherapy	Response	Prognosis
Bertoni *et al*. (2005) [3]	6	Humerus, femur, tibia	Resection	Details unknown	None	Grade II (50–89% necrosis: Huvos grading system)	NED: 4 DOD: 2
Sheth *et al*. (1996) [4]	10	Femur, tibia	En bloc excision	Intra-arterial cisplatin (dose range, 120–160 mg/m^2^), intravenously administered doxorubicin (dose range,60–90 mg/m^2^), 4.2 cycles (range, 2–12 cycles)	One of the following: ▪high-dose methotrexate ▪doxorubicin, dacarbazine ▪high-dose methotrexate, cisplatin ▪high-dose methotrexate, ifosfamide ▪high-dose methotrexate, doxorubicin, dacarbazine Doxorubicin, cisplatin	Good response: 4 (with > 90% necrosis of the high-grade component). Poor response: 6 (with < 90% necrosis of the high-grade component)	NED: 5 DOD: 5
Futani *et al.* (2001) [11]	1	Fibula	En bloc excision	Intravenously administered high-dose methotrexate (dose range, 9.5–11.4 g/m^2^), intra-arterial pirarubicin (dose range, 50–80 mg/m^2^), and dacarbazine (dose range, 400–600 mg/m^2^) three cycles	None	90% necrosis	NED

*DOD* dead of disease, *NED* no evidence of disease

## Conclusions

A rare case of DPOS in the maxilla is presented. Our report suggests that DPOS can arise from the head and neck region and chemotherapy consisting of THP-ADM and IFO could be effective for high-grade components. Further studies are needed to establish an appropriate treatment strategy for DPOS.

## Abbreviations

CDDP: Cisplatin
c-POS: Conventional low-grade parosteal osteosarcoma
CT: Computed tomography
DPOS: Dedifferentiated parosteal osteosarcoma
IFO: Ifosfamide
MRI: Magnetic resonance imaging
NAC: Neoadjuvant chemotherapy
THP-ADM: Pirarubicin
